# Geographical variation in colour of female threespine stickleback (*Gasterosteus aculeatus*)

**DOI:** 10.7717/peerj.4807

**Published:** 2018-05-16

**Authors:** Connor M. French, Travis Ingram, Daniel I. Bolnick

**Affiliations:** 1 Department of Zoology, Cooperative Wildlife Research Laboratory, Southern Illinois University at Carbondale, Carbondale, IL, USA; 2 Department of Integrative Biology, University of Texas at Austin, Austin, TX, USA; 3 Department of Zoology, University of Otago, Dunedin, New Zealand

**Keywords:** Female, Colour, Selection, Stickleback, Geography

## Abstract

The ecological multifunctionality of colour often results in multiple selective pressures operating on a single trait. Most research on colour evolution focuses on males because they are the most conspicuous sex in most species. This bias can limit inferences about the ecological drivers of colour evolution. For example, little is known about population divergence in colour of female threespine stickleback (*Gasterosteus aculeatus*), which is among the most intensively-studied model vertebrates in evolution, ecology, and behaviour. In contrast, the evolution and ecology of colour in male stickleback has received considerable attention. One aspect of female colouration that is lacking previous research is non-ornamental body colour. Non-ornamental colour can play defensive and social roles, and indicate other aspects of female stickleback ecology. To remedy this knowledge gap, we measured the colour and brightness of one dorsal and one ventral lateral area on female stickleback from nine lake populations on Vancouver Island. We found that lake populations varied in overall colour brightness and dorso-ventral contrast. In addition, we found that female brightness increased with lake size, indicating potential ecological drivers of these colour differences. Our results demonstrate that there is substantial scope for future research on female colour diversification, which has been overlooked because past researchers focused on dramatic male nuptial colours.

## Introduction

Intraspecific variation in colouration is common across the animal kingdom (Mallet & Joron, 1999; Gray & McKinnon, 2007; Protas & Patel, 2008; Seehausen et al., 2008). Colouration can play many different ecological roles, including crypsis, mimicry, aposematism, thermoregulation, and communication. The multifunctional nature of colouration often results in multiple selective pressures acting on the same trait (Cummings & Crothers, 2013). When ecological conditions vary spatially, this can lead to geographic variation in colour (Amundsen, 2000). Most research on among-population colour differences has focused on males of sexually dimorphic species, because males are usually the more conspicuous sex (Baker & Parker, 1979; Protas & Patel, 2008). Comparatively little attention has been given to geographic variation in females, the more generally, less-conspicuous sex. While there are many examples of female ornamentation and colour variation, the evolution and ecology of female colour is still poorly understood (Amundsen, 2000; Nordeide et al., 2013).

The threespine stickleback (*Gasterosteus aculeatus*) is a good system for investigating the evolution of colour due to its high intraspecific phenotypic diversity and the abundance of research on its ecology (Wootton, 1984; Schluter, 2001; McKinnon & Rundle, 2002; Östlund-Nilsson, Mayer & Huntingford, 2006) and courtship behaviour (Candolin, 1997; Ishikawa & Mori, 2000). Populations of stickleback are often fully or partially isolated by lakes, whose size can reflect distinct community composition and trophic relationships (Wellborn, Skelly & Werner, 1996; Tessier & Woodruff, 2002). Predator diversity and predation pressure vary with community composition (Wellborn, Skelly & Werner, 1996; Braoudakis & Jackson, 2016). This variation can differentially impact the evolution of prey defence mechanisms, such as crypsis (Husak et al., 2006). Additionally, there is a wealth of information on colour diversity of male stickleback between and within populations (Brock, Cummings & Bolnick, 2017; Reimchen, 1989; Milinski & Bakker, 1990; Bakker, 1993; Boughman, 2001, 2007; Boughman, Rundle & Schluter, 2005). Given this large body of research on male stickleback colour, it is remarkable how little is known about female colour in stickleback. There are stream-resident populations of stickleback where females have red throats similar to male nuptial colouration (McKinnon et al., 2000; Yong et al., 2013). The genetic architecture of red throat colouration is shared between males and females, but its adaptive function, if there is any, in females is still uncertain (Yong et al., 2013, 2015; Yong, Peichel & McKinnon, 2016; Wright et al., 2015). In addition, conspicuous red pelvic spines have been observed in North American and European populations of threespine stickleback (Amundsen et al., 2015; Yong, Peichel & McKinnon, 2016). There is no genetic component to pelvic spine colouration in European populations, and its potential adaptive function is also uncertain (Amundsen et al., 2015). Other more general female colour patterns include barring and mottling in both the human visible and ultraviolet UV spectra (Rowland, Baube & Horan, 1991; Rick & Bakker, 2008). Experiments show that these patterns indicate female sexual receptiveness (Rowland, Baube & Horan, 1991; Rick & Bakker, 2008). However, between-population differences in non-ornamental female colour remain largely unknown. To remedy this surprising lacuna, here we present evidence that female threespine stickleback exhibit substantial body colour variation among nine lakes on Vancouver Island, British Columbia.

## Methods

**Figure 1:**
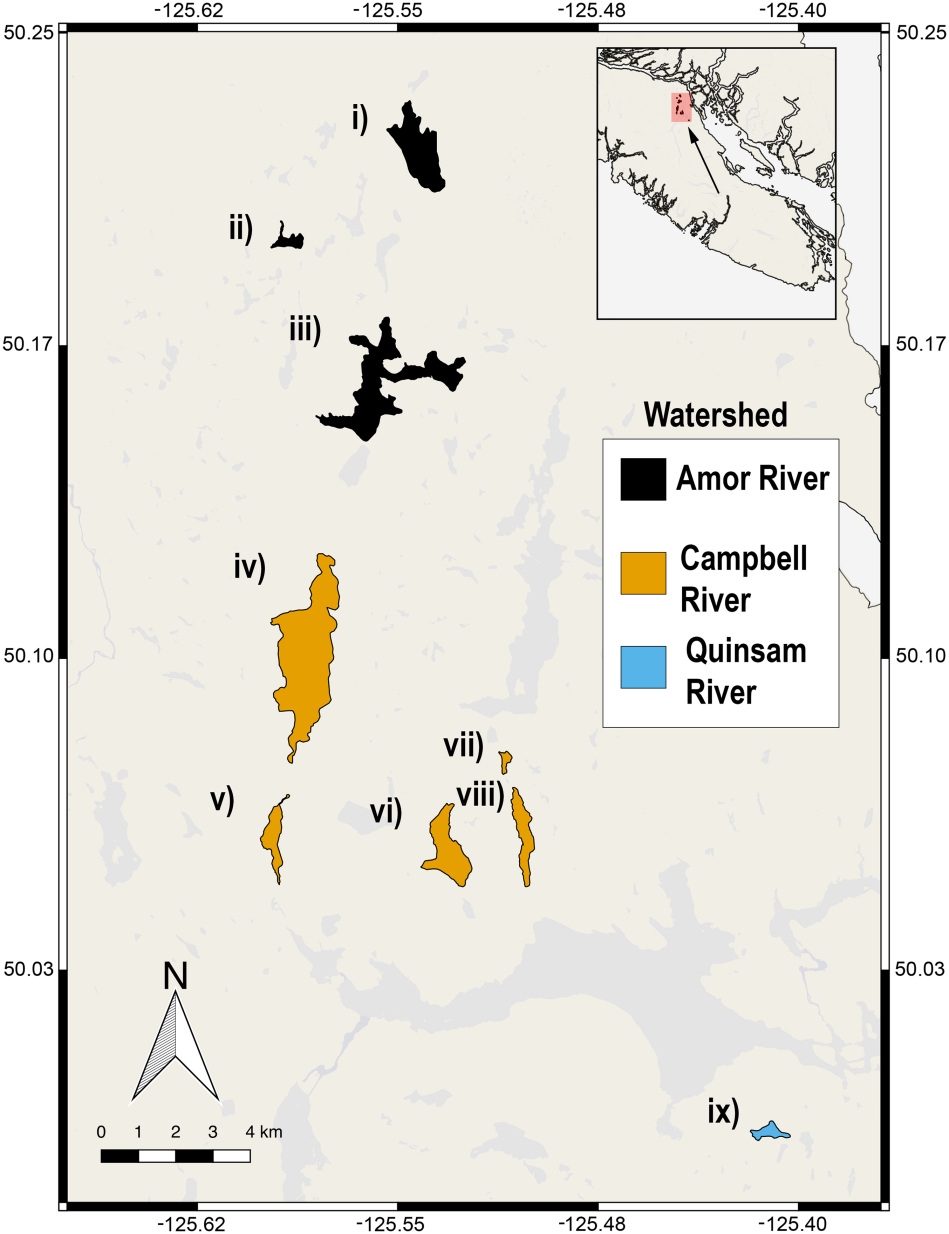
Nine lake populations from Vancouver Island, British Columbia (inset) from which stickleback populations were sampled. Lakes are coloured according to their watershed (see legend). The sampled lakes are: (i) Roberts Lake (*n* = 10; SA = 160.0 ha), (ii) Farewell Lake (*n* = 10; SA = 20.3 ha), (iii) Amor Lake (*n* = 10; SA = 329.9 ha), (iv) Brewster Lake (*n* = 20; SA = 470 ha), (v) Grey Lake (*n* = 10; SA = 52.9 ha), (vi) Boot Lake (*n* = 2; SA = 98.7 ha), (vii) Echo Lake (*n* = 6; SA = 20.6 ha), (viii) Gosling Lake (*n* = 9; SA = 62.5 ha), (ix) Higgens Lake (*n* = 5; SA = 10.6 ha), where ‘*n*’ is the number of fish sampled and ‘SA’ is the surface area of the lake.

We captured a total of 82 female threespine stickleback from nine lakes present in three watersheds on Vancouver Island, British Columbia (Fig. 1). We placed 10–20 unbaited Gee minnow traps in each lake between late May and mid-June 2013, in depths from 50 to 300 cm. Traps were set in the morning between 09:30 and 10:30, and were retrieved between 16:00 and 18:00. The fish were transported to shore in clear plastic containers filled with lake water, immediately after removal from the traps. A maximum of five fish were transported in a given container to mitigate stress to the animal and reduce the amount of colour change that may result from stress. The total time from retrieval to photograph was less than 15 min. We noted the reproductive state (gravid or not gravid) of each fish, since sampling was performed during their spawning season. Collections were carried out with approval from the University of Texas at Austin Institutional Animal Use and Care Committee AUP-2011-00044, and with a Scientific Fish Collection permit from the Ministry of Forests, Lands and Natural Resource Operations in British Columbia NA12-84188.

Captured fish were placed unanesthetized in an empty 11 L cubic cooler with the inside spray-painted matte black to reduce glare. They were placed right side up on a light blue rubber dissecting mat with a colour card (CameraTrax; Menlo Park, California) attached for colour standardization. A Fotodiox Pro LED-209A light system was fastened to the inside wall of the cooler and was kept parallel to the specimen at the most powerful light setting to prevent shadowing and uneven lighting (the light output does not fade with battery power decline). A Canon Rebel 3i DSLR camera was positioned in a hole in the cooler lid directly over the specimen. The camera was set to an F-stop of 7.1, a shutter speed of 1/125, and white balance was set to auto. Images were 18 megapixel JPEGs. White balance standardization was completed in Adobe Photoshop CC^©^ using the Curves tool, where the colour card was used as a reference. One photograph was taken per specimen.

**Figure 2:**
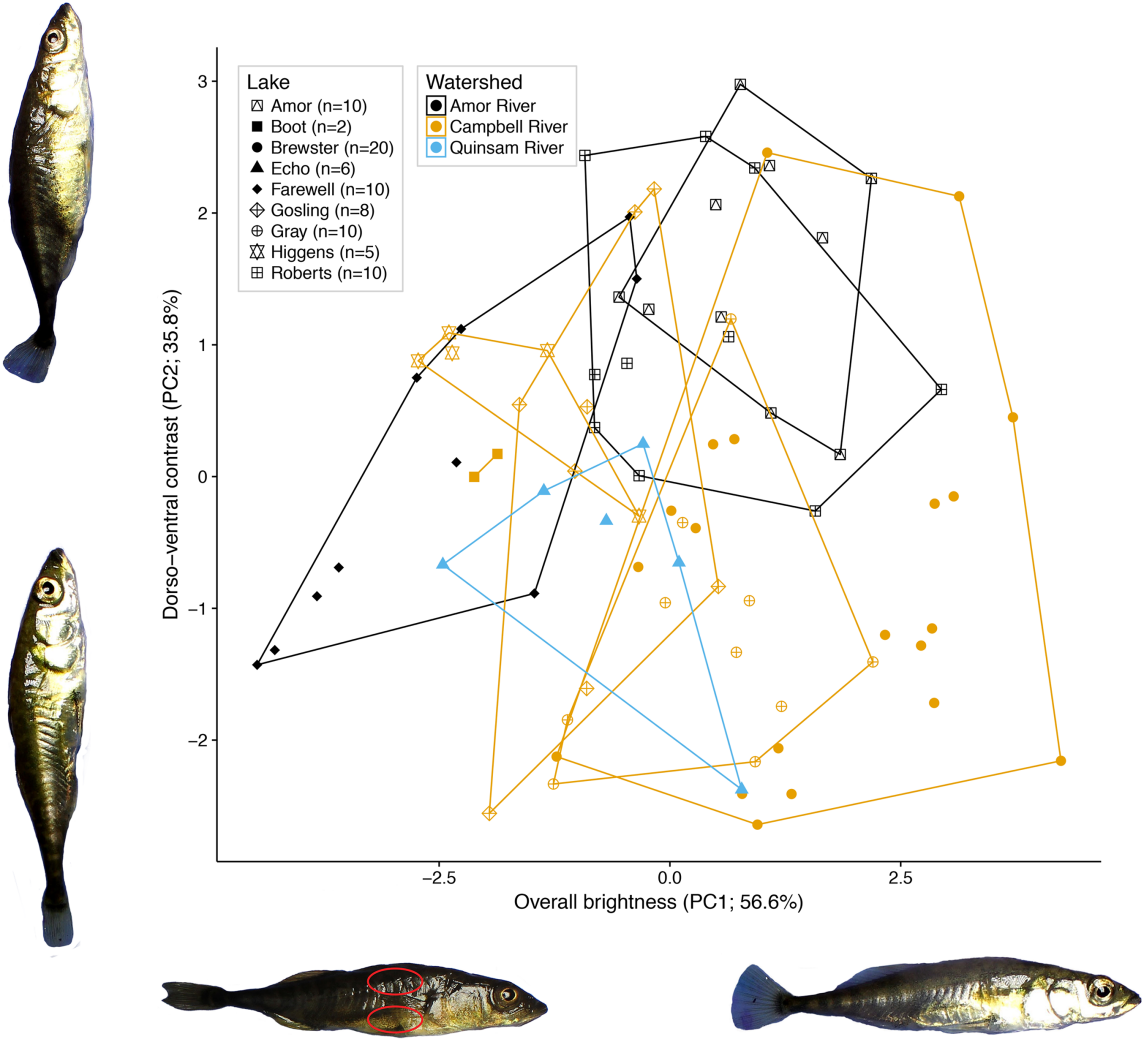
Variation in female colour among nine lake populations from Vancouver Island, British Columbia. The axes represent colour as measured by the first and second PCs calculated from red, green, and blue brightness values from dorsal and ventral locations on each female. The two major axes, plotted here, represent overall colour brightness (PC1) and dorso-ventral contrast (PC2). The percent variances attributed to PC1 and PC2 are listed along each corresponding axis. Lakes are distinguished by different point shapes and a bounding convex hull, and watersheds are distinguished by different colours (see legend). Photographs of fish representative of extreme PC values are included to illustrate female colour differences. One fish is annotated with two red ovals, indicating the areas measured for colour analysis. Photography credit: Connor M. French.

We obtained RGB brightness levels averaged from the area inside two small ovals per fish, each approximately 5 mm in diameter, one ventral to the dorsal fin and one posterior to the pectoral fin (Fig. 2). These areas were chosen to reflect dorsal and ventral non-ornamental body colour variation, respectively. Measurements were made using Image J (Schneider, Rasband & Eliceiri, 2012). We performed a principal components analysis on the RGB brightness level matrix to obtain composite RGB brightness variables across all photographed specimens.

We digitally measured lake surface area from Google Earth^©^. We cross-checked our measurements against the British Columbia Ministry of Environment HabitatWizard database values, which were available for about two-thirds of the lakes, to confirm a high correlation. We performed a nested fixed-effects multivariate analysis of variance (MANOVA) to test for differences in fish colour across lakes nested within watersheds. Our data conformed to the parametric assumption of multivariate normality (Royston’s Multivariate Normality Test; *P* > 0.05), yet our data did not conform to the assumption of multivariate homogeneity of variance (Box’s *M*-test for Homogeneity of Covariance; *P* < 0.01). However, we proceeded with parametric analyses since Box’s *M*-test is highly sensitive to slight deviations from normality and MANOVA tends to be robust to violations of this assumption. We retained the first two PC axes, which explained 92.4% of the variation in colour. We used the two retained PC scores as dependent variables, and lake identity nested within watershed as independent variables. We initially included date of capture and reproductive state (gravid versus not gravid) as covariates, but both were nonsignificant (*P* > 0.05) and were left out of the final model. We performed follow-up nested fixed-effects analyses of variance (ANOVA) for each PC axis to see how the colour variables differed across lakes nested within watershed. To address possible concerns about low sample size per lake, we repeated these analyses, focusing only on the five lakes for which we measured at least ten females.

We analysed the relationship between lake surface area and both colour PC scores using weighted linear regression, where we weighted the median PC score for each lake by the square root of its sample size. Considering the small number of populations overall, we focused on this single well-defined predictor of known ecological importance. We should note that we considered lake turbidity as a covariate, as the light environment is known to influence stickleback colouration (Lewandowski & Boughman, 2008), but measurements could not be made for all lakes. We therefore excluded lake turbidity from analysis. Our linear models met all assumptions. All analyses were performed using R v.3.3.2 (R Core Team, 2016).

## Results

Of the 92.4% of variation retained by the first two principal components, the first PC (56.6% of the variation) represents total colour brightness, as all six colour variables load negatively on PC1 (Fig. 2). The second PC (35.8% of variation) represents dorso-ventral contrast, as dorsal RGB values load positively on PC2, while ventral RGB values load negatively.

A nested fixed-effects MANOVA of individuals’ PC1 and PC2 scores revealed significant differences in colour across watersheds (*F*_2, 72_ = 9.70, *P* < 0.001) and lakes nested within watersheds (*F*_6, 72_ = 9.00; *P* < 0.001; Fig. 2) for the nine populations. Watershed and lake identity nested within watershed also had significant effects on PC1 and PC2 analysed in separate ANOVAs after multiple test correction (PC1: adjusted *P* = 0.007; PC2: adjusted *P* < 0.001; Benjamini & Hochberg, 1995). The results for the reduced dataset (five lakes with *N* > 10 each) were largely similar. There was significant variation in colour across watersheds (*F*_1, 55_ = 36.915, *P* < 0.001) and lakes nested within watersheds (*F*_3, 55_ = 6.818; *P* < 0.001) for the five lakes. Additional PC axes explained relatively little colour variance, where no single axis explained more than five percent of the variance.

**Figure 3:**
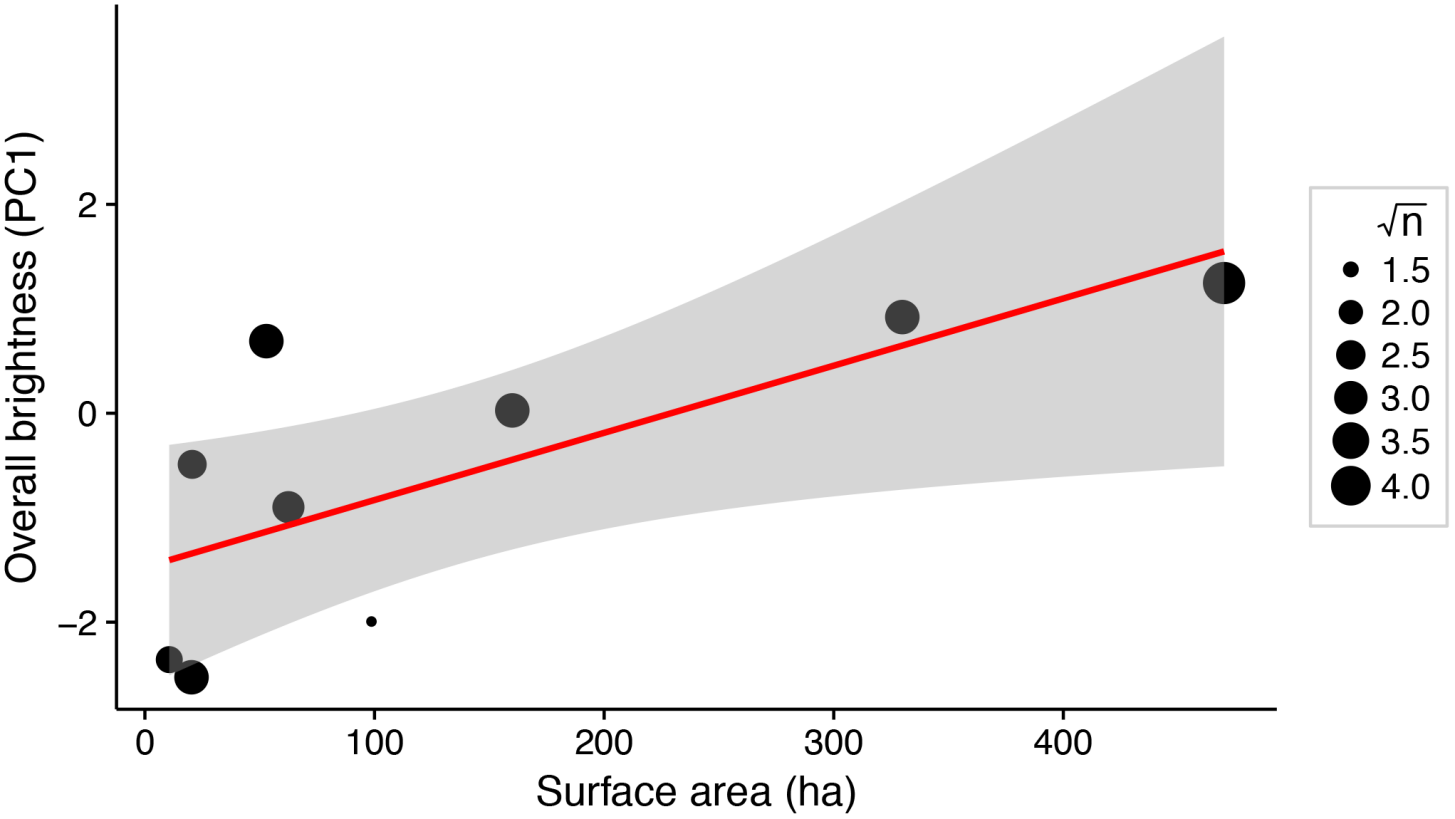
Weighted linear regression between lake surface area (ha) and overall brightness of female stickleback (median PC1 scores for each lake). Data points are weighted by the square root of the sample size for each lake (√n). Shading around the regression line indicates a 95% confidence interval.

There was a significant positive relationship between lake surface area and female brightness (*R*^2^ = 0.56; *P* = 0.02; Fig. 3), but not between lake surface area and dorso-ventral contrast (*R*^2^ < 0.001; *P* = 0.97).

## Discussion

A multifunctional trait like colour is likely acted on by multiple selective pressures (Gray & McKinnon, 2007; Protas & Patel, 2008). Selective pressures vary across heterogeneous environments, so colour variation is expected among geographically separated populations (Hoekstra, 2006). Many of these selective pressures will apply to both sexes, rather than just the sex that experiences particularly strong sexual selection. We therefore expect that, in sexually dimorphic species, female colour will also vary among populations in response to environmental variables and selective forces such as predation favouring crypsis. In line with this expectation, we found that female threespine stickleback vary in body colour across lake populations and among watersheds in a small geographic area (Fig. 2). This variation entailed population differences in both overall brightness and dorso-ventral contrast. It is not known at present to what extent this observed variation, which was measured on freshly wild-caught fish, is heritable or environmentally induced. Among female stickleback, dorsolateral barring patterns signal sexual receptiveness (Rowland, Baube & Horan, 1991). Males prefer enhanced UV contrast between barring patterns and their silvery flank, suggesting sexual selection for female colour at human visible and UV wavelengths (Rick & Bakker, 2008; Hiermes et al., 2015). UV intensity and barring contrast increase as females become gravid (Rowland, Baube & Horan, 1991; Rick & Bakker, 2008; Hiermes et al., 2015), but we found that reproductive state does not influence between-lake colour variation in our system.

Female red throat colour, an ornamental trait analogous to male red throat colour, has a genetic component (Yong, Peichel & McKinnon, 2016). The genetic architecture is shared between male and female red ornaments, but they seem to be differentially expressed (Yong, Peichel & McKinnon, 2016). There is some evidence that red throated females from a single stream population grow faster than unornamented females (Yong et al., 2013). However, red throat colour does not indicate female receptiveness, is not associated with a competitive advantage, and is not preferentially selected for by males (McKinnon et al., 2000; Yong et al., 2013, 2015; Wright et al., 2015). The function of female red pelvic spines is similarly ambiguous (Amundsen et al., 2015). In contrast to these studies of ornamental colouration, we provide some evidence for the adaptive potential of non-ornamental female colour.

Predator assemblages vary with lake size, so variation in predation regime across lakes of different sizes may contribute to variation in female colour (Reimchen, 1994). We show that total body colour brightness increases with lake surface area (Fig. 3). A somewhat similar pattern is seen in male stickleback, whose conspicuousness generally increases with lake depth (Brock, Cummings & Bolnick, 2017). Predator regime differed between the lakes’ benthic and limnetic environments, although the trend was not significant (Bolnick et al., 2016; Brock, Cummings & Bolnick, 2017). In addition, increased lake size is associated with morphological traits suited to zooplanktivory across stickleback populations (Lavin & McPhail, 1985). The associated difference in diet, or affiliated parasite exposure, could affect sticklebacks’ condition and nutrition in ways that could alter female colour. Diet and parasite exposure are already known to influence the colour of male stickleback (Milinski & Bakker, 1990; Milinski, 2003; Pike et al., 2007, 2009). Further inquiry into the selective consequences of predation and trophic specialisation for female stickleback colour may reveal insight about the factors driving their colouration divergence.

## Conclusion

This study reveals variation in female stickleback colouration across populations, associated with a major ecological gradient (lake size). Our findings justify future inquiry into the mechanistic physiological, behavioural, and ecological causes of this variation. There has been a recent increase in interest in female stickleback colouration (Rick & Bakker, 2008; Yong et al., 2013, 2015; Yong, Peichel & McKinnon, 2016; Wright et al., 2015; Hiermes et al., 2015). In addition to these recent studies, our results suggest that female stickleback are an opportune study system for understanding colour evolution. By combining male and female colour variation into a single study, future researchers may be better able to distinguish population differences arising from divergent sexual versus natural selection. More generally, our finding of strong population divergence in female colour serves as a reminder that colour is a multi-purpose phenotype that is under selection in both sexes, and that researchers should avoid focusing on male colour evolution alone.

## Supplemental Information

10.7717/peerj.4807/supp-1 Supplemental Information 1 Dataset of colour measurements, geographic information, time, and reproductive state. This is the total dataset, including field number, lake designation, raw RGB brightness levels, lake surface area in hectares, watershed designation, date (in month/day format), and gravidity designation, where 1 is gravid, and 0 is not gravid.

## Additional Information and Declarations

### Field Study Permissions

The following information was supplied relating to field study approvals (i.e. approving body and any reference numbers): Field experiments were approved by the Ministry of Forests, Lands and Natural Resource Operations in British Columbia (Scientific Fish Collection Permit).

### Data Availability

The following information was supplied regarding data availability: The raw dataset is provided as a Supplemental File.
